# Cognitive outcomes and psychological symptoms in an Italian cohort with post-acute COVID-19 condition (PACC)

**DOI:** 10.1016/j.heliyon.2024.e39431

**Published:** 2024-10-16

**Authors:** Alessandra Vergori, Giulia Del Duca, Paola Borrelli, Anna Clelia Brita, Carmela Pinnetti, Ilaria Mastrorosa, Marta Camici, Annalisa Mondi, Valentina Mazzotta, Pierangelo Chinello, Paola Mencarini, Maria Letizia Giancola, Amina Abdeddaim, Enrico Girardi, Andrea Antinori

**Affiliations:** a Viral Immunodeficiency Unit, National Institute for Infectious Diseases Lazzaro Spallanzani IRCCS, Rome, Italy; b Psychology Unit, National Institute for Infectious Diseases Lazzaro Spallanzani IRCCS, Rome, Italy; c Laboratory of Biostatistics, Department of Medical, Oral and Biotechnological Sciences, University “G. D'Annunzio” Chieti-Pescara, Chieti, Italy; d Severe and Immune-Depression Associated Infectious Diseases Unit, National Institute for Infectious Diseases Lazzaro Spallanzani IRCCS, Rome, Italy; e Respiratory Infectious Diseases Unit, National Institute for Infectious Diseases Lazzaro Spallanzani IRCCS, Rome, Italy; f Emerging Infectious Diseases Unit, National Institute for Infectious Diseases Lazzaro Spallanzani IRCCS, Rome, Italy; g Hepatology Unit, National Institute for Infectious Diseases Lazzaro Spallanzani IRCCS, Rome, Italy; h Scientific Direction, National Institute for Infectious Diseases Lazzaro Spallanzani IRCCS, Rome, Italy

**Keywords:** Post-COVID19, Long-COVID-19, Cognitive outcomes, Neuropsychological symptoms, Sleep disorders

## Abstract

**Background:**

We aim to investigate the proportion of patients (pts) with long-term cognitive outcomes (CO) of PACC and identify associated features.

**Methods:**

We assessed participants through a neuropsychological assessment. The chi-square test was used for comparisons according with time of NPA (within or beyond 6 months since COVID19) and with previously hospitalization status (hospitalized patients, PH; not hospitalized patients, nPH).

**Results:**

520 participants: mean age 54 years (SD 12), 53 % female, 14 years of education (SD 3.4), 35 % with >1 comorbidity, 48 % previously hospitalized. Overall, we found CO in 89 % of pts, in particular 88 % evaluated in w6M and 89 % in b6M (p = 0.801) while 90 % and 87 % in nPH and PH, respectively (p = 0.239). By fitting multivariable analysis, PH for COVID19 and female gender were associated with an increased risk of an altered PSQI [Odd Ratio, OR 2.48, 95 % CI 1.54 to 3.99, p < 0.001 and OR 2.59, 95 % CI 1.60 to 4.17, p < 0.001, respectively) and BAI [F vs M: OR 1.67, 95 % CI 1.16 to 2.40, p = 0.005).

**Conclusions:**

We show a substantial proportion of PACC-CO; hospitalization leads to impaired memory, anxiety and sleep disorders. Women seem to be at higher risk for anxious-depressive symptoms and worse sleep quality than men.

## Highlights

• Cognitive impairment can occur in patients without subjective complaints.
• COVID-19 can lead to lasting neurocognitive issues.
• All patients with PACC should undergo neurocognitive assessments.

## 1 Background

The post-acute COVID condition (PACC) is characterized by a range of recurring or persistent symptoms experienced after infection with SARS-CoV-2, lasting for several weeks following the onset of COVID-19 and with uncertain outcomes [1]. These symptoms include psychiatric manifestations [2] and neurocognitive impairment (NCI) [[3], [4], [5]]. NCI is among the most common prolonged symptoms of COVID-19, as outlined by the WHO consensus on post-COVID-19 condition [6,7]. Two meta-analyses have estimated the overall prevalence of cognitive problems to be between 14 % and 22 % [8,9], while the incidence has ranged from 0 % to 78 % (both in the acute and post-acute COVID-19 phases) [10], with significant variability due to the diversity of study populations and neuropsychological assessments. Hospitalization for COVID-19 appears to be a risk factor for more pronounced cognitive disorders [11]. However, persistent cognitive and psychological symptoms may also be present in non-hospitalized individuals [[12], [13], [14]], including younger non-hospitalized patients with mild to moderate infections [[15], [16], [17]].

Moreover, cognitive assessments could be considered “objective” when conducted through tests administered by trained examiners [18] or “subjective” when based on self-reported questionnaires [19,20]. Different rates of impairment are observed when comparing objective and subjective measures, indicating that many individuals with cognitive disorders may not be aware of or report their condition, while others with cognitive complaints may not have objectively measurable cognitive disorders [[21], [22], [23]].

The primary aim of our study was to investigate the proportion of individuals with post-acute COVID-19 cognitive outcomes (PACC-CO) in an Italian cohort of patients with or without cognitive complaints. The secondary aims were to characterize the type of cognitive deficits based on test performance and to identify features associated with PACC-CO.

## 2 Methods

### 2.1 Study design, setting and study population

This is an observational, retrospective, monocentric study conducted at the post-COVID services of the National Institute for Infectious Diseases "L. Spallanzani" in Rome, Italy (Neuro-COVID Study).

Inclusion criteria for enrollment included patients with a documented history of SARS-CoV-2 infection (evidenced by the presence of SARS-CoV-2 RNA in a biological specimen or positive serology, along with compatible clinical/pulmonary findings) and neurological signs or symptoms. These patients were consecutively followed up at the Clinical Department of the National Institute for Infectious Diseases "L. Spallanzani" in Rome. Key data on blood tests, radiological examinations, neurological symptoms and signs, medical history, and treatments received were collected retrospectively from medical records.

Patients, regardless of neuropsychological complaints, underwent neurocognitive screening, imaging studies, and, if not contraindicated, diagnostic lumbar puncture, where and when possible. All tests were administered based on the patient's clinical condition and the attending physician's judgment, with potential follow-ups scheduled after 1–3 months.

The objectives of the main Neuro-COVID study and its sub-studies were: a) to evaluate the presence and progression of neurological signs and symptoms in SARS-CoV-2-infected patients over time; b) to assess the neurocognitive status of patients during both acute and post-acute phases using a comprehensive battery of tests covering five domains; c) to describe neuroimaging findings through CT perfusion studies and/or brain MRI with functional analysis; d) to explore virological and immunological correlations; and e) to examine associations between psychopathological symptoms (such as anxiety, depression, and sleep disorders) and neurocognitive status. Patients had the option to choose which sub-studies to participate in.

This analysis focuses on the subset of patients who opted to take part in the neurocognitive assessment sub-study between September 2020 and November 2022, following the acute phase of COVID-19. We included patients referred to our post-COVID-19 service, evaluated within and beyond six months of testing positive for SARS-CoV-2 via PCR, both previously hospitalized and non-hospitalized for COVID-19, and referred by a clinician or self-referred to our post-COVID-19 outpatient service for symptoms attributable to PACC. These evaluations took place at least four weeks after the acute SARS-CoV-2 infection, as part of a neuropsychological screening program during the post-acute phase of COVID-19.

### 2.2 Study procedures

Epidemiological, demographic data and clinical data were collected and anonymously recorded. Neuropsychological assessment (NPA) was performed using a standardized battery of 20 tests across 5 cognitive domains (memory, attention, language, executive functions, speed of psycho-motor processing). PACC-CO was classified based on the scores into equivalent scores (ES) in each test and defined by the presence of alterations (<cutoff-ES = 0) in at least one test. ES makes it possible to establish the test subject's position relative to normal subjects, net of the influence of variables related to gender, age and education. Depending on the test and the basic demographic variables (gender, age and education), the raw score obtained by the test subject is adjusted and then converted into the corresponding ES, classified on a 5-level scale: ES = 0 Deficient comprising; ES = 1 Borderline performance; ES = 2–3 Middle-lower comprising performance; ES = 4 Middle-upper comprising performance. All the references related with each test and insights are reported in supplementary methods.

### 2.3 Statistical analysis

Descriptive analysis was carried out using mean and standard deviation (SD) or median and interquartile range (IQR) for the continuous variables and percentages values for the categorical ones. Normality distribution for continuous variables was assessed by the Shapiro-Wilk Test. Pearson's chi-square test or Fisher's exact test was used to evaluate the association between categorical variables while the independent samples T test or the analogous non-parametric Wilcoxon rank-sum test between continuous variables and outcome considered.

Crude odds ratio (ORs) and corresponding 95 % CI were calculated in order to quantify the risk associated between explicative variables (socio-demographic and clinical features) and endpoint variables (neuropsychological alteration test) using the Wald test. Subsequently, single multivariable logistics regression models were done to identify the association between each endpoints considered (dependent variables: ROCF-Copy, Categorical Verbal Fluency, BAI>85 %, RAVLT-DR, ROCF-DR, Corsi Span Forward, BDI-II>85 % and PSQI>5 categorized as 0 = no altered, 1 = altered, respectively) and the independent variables found significant in univariate analysis, in absence of collinearity and contributive to the model fit (Likelihood Ratio test): gender, age, time within and beyond 6 months since the SARS-CoV-2 PCR test positivity after acute COVID-19 and previously hospitalized or not previously hospitalized for COVID-19, respectively, Statistical significance was set at the level of ≤0.05. All analyses were performed using Stata software v17.1 (StataCorp, College Station, USA).

## 3 Ethics

Neuro-COVID study was approved by the Ethics Committee of ‘INMI Lazzaro Spallanzani’, according to the Italian legislation for SARS-CoV-2 studies (approval number 119/2020 of May 20, 2020). The study was conducted in accordance with the Declaration of Helsinki. An informed consent was obtained from all subjects involved.

## 4 Role of the funding source

This research did not receive any specific grant from funding agencies in the public, commercial, or not-for-profit sectors.

## 5 Results

### 5.1 Population characteristics

The analysis included N = 520 patients with a mean age of 54 years (Standard Deviation, SD ± 12), of whom 54 % were female, with an average of 14 years of education (SD ± 3.4). Additionally, 35 % had at least one comorbidity, and 48 % had been previously hospitalized for acute COVID-19. Among these hospitalized patients, 36 % received oxygen therapy: 6 % with low-flow oxygen, 15 % with high-flow oxygen via Venturi-Mask, 7 % required non-invasive ventilation (NIV), and only 2 patients underwent oro-tracheal intubation (OTI). The general characteristics of the study population are summarized in Table 1.

**Table 1:** Demographic and clinical characteristic of participants at baseline and by groups (not previously hospitalized vs previously hospitalized (nPH vs PH)).

Variables	Total (N = 520)	nPH (N = 271)	PH (N = 249)	ap-value
Age, mean (SD)	53.8 (12)	50.7 (11.9)	57.3 (11.1)	<0.0001
Female, No. (%)	279 (53.6)	160 (59.0)	119 (47.8)	0.010
Education, mean (SD)	13.9 (3.4)	14.4 (3.0)	13.3 (3.7)	<0.0001
Time since COVID-19 in days, mean (SD)	245 (158.5)	244.1 (157.4)	246.5(160.0)	0.859
BMI, mean (SD)	26.1 (4.9)	24.8 (4.4)	27.5 (5.1)	<0.0001
At least 1 comorbidity, No.(%)	183 (35.2)	77 (28.4)	106 (42.6)	0.001
Comorbidities, No. (%)				
*Hypertension*	151 (29.2)	206 (76.0)	161 (65.2)	0.007
*Diabetes*	43 (8.3)	16 (5.9)	27 (10.9)	0.038
*Cardiac Disease*	17 (3.3)	4 (1.5)	13 (2.5)	0.072
*Neurological disease*	10 (1.9)	2 (0.7)	8 (3.2)	0.054
*COPD*	14 (2.7)	6 (2.2)	8 (3.3)	0.590
*Cancer*	5 (1.0)	2 (0.7)	3 (1.2)	0.673
ICU admission, No. (%)	16 (3.2)	1 (0.4)	15 (6.7)	<0.0001
Oxygen therapy, No. (%)	185 (35.8)	8 (2.3)	177 (71.9)	<0.0001
TEP, No. (%)	19 (4.1)	1 (0.4)	18 (8.5)	<0.0001
Anti-SARS-CoV-2 therapy, No. (%)				
*Convalescent Plasma*	4 (1.5)	0 (0.0)	4 (3.6)	0.028
*Tocilizumab/sarilumab*	8 (1.6)	0 (0.0)	8 (3.5)	0.002
*Kaletra*	111 (22.2)	34 (12.6)	77 (33.3)	<0.0001
*Steroids*	253 (50.7)	95 (35.1)	158 (69.3)	<0.0001
*Remdesivir*	81 (16.4)	2 (0.7)	79 (35.3)	<0.0001
*Hydroxychloroquine*	79 (15.9)	14 (5.2)	65 (28.6)	<0.0001
*LWMH prophylaxis*	152 (29.2)	39 (7.5)	132 (25.4)	<0.0001

Abbreviations: BMI, Body Mass Index; COPD, Chronic Obstructive Pulmonary Disease, ICU, Intensive Care Unit; Low weight molecular hepatin; nPH, not Previously Hospitalized for COVID19; Previously Hospitalized for COVID19.

a p-values are for Student T test for independent data; Pearson's chi-square test or Fisher's exact test.

Briefly, as expected, the previously hospitalized (PH) group was significantly older than the non-hospitalized (nPH) group, with higher body mass index (BMI) and a greater proportion of patients with diabetes. A higher percentage of previously hospitalized patients were admitted to the Intensive Care Unit (ICU) and received supplemental oxygen therapy compared to nPH patients. Most of the study population belonged to the first two pandemic waves, a period when therapeutic options were limited. Consequently, a higher proportion of previously hospitalized patients (vs. not previously hospitalized) received treatments such as Lopinavir/ritonavir (33 % vs. 12 %, p < 0.0001) and Hydroxychloroquine (28 % vs. 5 %, p < 0.0001), in addition to steroid therapy and prophylaxis with low molecular weight heparin (LMWH) (Table 1, Supplementary Methods).

Of the total population, 7 % (36/520) were evaluated in 2020, 61.5 % (320/520) in 2021, and 31.5 % (164/520) in 2022. Overall, according to our definition, a cognitive outcome (CO) was found in 89 % of patients, with no significant differences between those evaluated within six months (w6m) and beyond six months (b6m) after acute COVID-19 (88 % vs. 89 %, p = 0.801), nor between not and previously hospitalized groups (90 % vs. 87 %, p = 0.239).

**Fig. 1:**
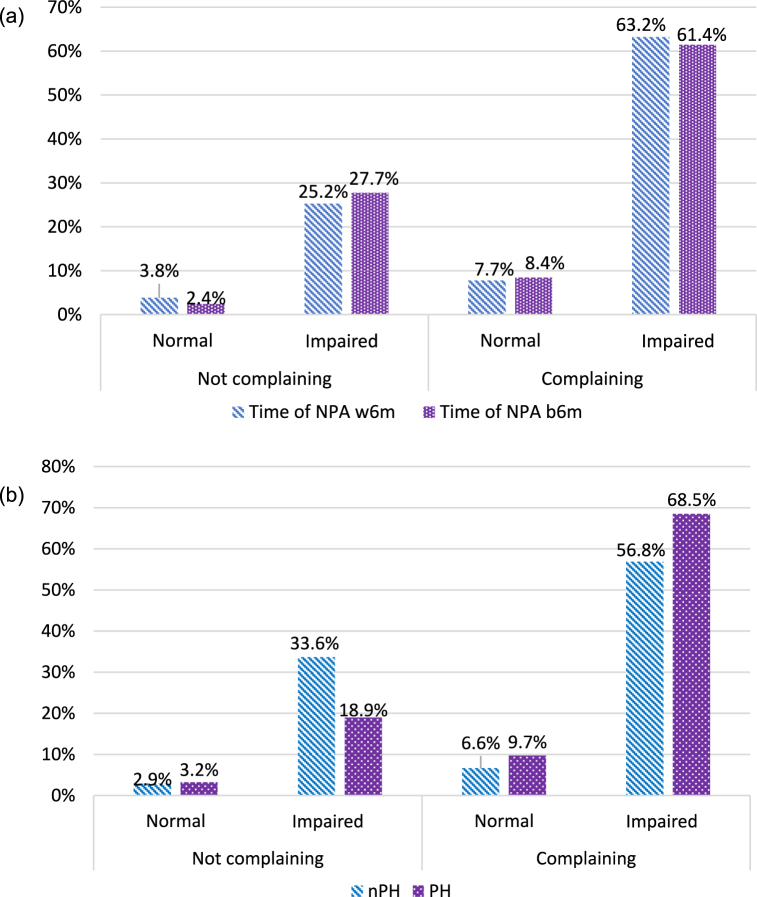
**A**. Proportions of normal and impaired neuropsychological performance compared with presence of symptoms (*complaining*), absence of symptoms (*not complaining*), neuropsychological assessment performed within 6 months from acute infection (*Time of NPA w6m*), after 6 months from acute infection (*Time of NPA b6m*) (Pearson chi2 = 1.263; p = 0.738).**B**. Proportions of normal and impaired neuropsychological performance compared with presence of symptoms (*complaining*), absence of symptoms (*not complaining*) and to hospitalization status (Pearson chi2 = 14.592; p = 0.002).

Notably, 90 % (138/154) of participants without cognitive complaints had at least one impaired cognitive test, compared to 88 % (323/365) of those reporting cognitive symptoms (p = 0.712). Among patients with cognitive complaints, a higher proportion had impaired test results, though no significant differences were observed in neuropsychological performance between those evaluated within 6 months and beyond (Fig. 1A). Conversely, according to hospitalization status, a significantly higher proportion of altered neuropsychological performances were found among previously hospitalized patients compared to those not previously hospitalized patients with cognitive complaints (Fig. 1B).

### 5.2 Analysis of impaired neurocognitive and psychological tests according to time of NPA evaluation

Analyzing neuropsychological assessments (NPA) in greater detail and by the time of evaluation, we observed the following proportions: 6.3 % of patients evaluated beyond 6 months had impaired performance on the Corsi Span Forward (CSF) test, compared to 1.3 % evaluated within 6 months (p = 0.004), 9.1 % had an impaired Corsi Span Backward (CSB) test versus (vs.) 3.4 % (p = 0.009). These findings suggest alterations in short-term memory, visuospatial capacity, and working memory, respectively.

Regarding psychological aspects, comparing evaluations performed within 6 months vs. those performed beyond 6 months, we found: Beck Anxiety Inventory (BAI) score >85 % in 40 % vs. 47 % (p = 0.123), as well as, the Beck Depression Inventory-II (BDI-II) score >85 % in 47 % vs. 46 % (p = 0.874). Interestingly, the Pittsburgh Sleep Quality Index (PSQI) > 5 was found in 11 % of patients vs. 31 % (p < 0.001), indicating a significant difference in sleep quality (Table 2; right panel).

**Table 2:** Normal and impaired neurocognitive and psychological tests according to time of NPA and previous hospitalization for COVID19.

*NEUROCOGNITIVE ASSESSMENT*	*Total*^*§*^	*nPH*	*PH*	∗p-value	*w6m*	*b6m*	∗p-value
	LEFT PANEL				RIGHT PANEL		
	N = 520	N = 271	N = 249		N = 234	N = 286	
*IADL median (IQR)*	8.0 (8.0–8.0)	8.0 (8.0–8.0)	8.0 (8.0–8.0)	0.247	8.0 (8.0–8.0)	8.0 (8.0–8.0)	0.346
*MMSE, No. (%)*							
**Normal**	513 (98.8 %)	270 (99.6 %)	243 (98.0 %)	0.108	233 (99.6 %)	280 (98.2 %)	0.230
**Impaired**	6 (1.2 %)	1 (0.4 %)	5 (2.0 %)		1 (0.4 %)	5 (1.8 %)	
*RAVLT-ST, No. (%)*							
**Normal**	459 (88.4 %)	247 (91.1 %)	212 (85.5 %)	0.044	207 (88.5 %)	252 (88.4 %)	0.989
**Impaired**	60 (11.6 %)	24 (8.9 %)	36 (14.5 %)		27 (11.5 %)	33 (11.6 %)	
*RAVLT-DR, No. (%)*							
**Normal**	472 (90.9 %)	246 (90.8 %)	226 (91.1 %)	0.888	214 (91.5 %)	258 (90.5 %)	0.714
**Impaired**	47 (9.1 %)	25 (9.2 %)	22 (8.9 %)		20 (8.5 %)	27 (9.5 %)	
*RAVLT-REC, No. (%)*							
**Normal**	415 (80.4 %)	230 (84.9 %)	185 (75.5 %)	0.007	185 (79.4 %)	230 (81.3 %)	0.594
**Impaired**	101 (19.6 %)	41 (15.1 %)	60 (24.5 %)		48 (20.6 %)	53 (18.7 %)	
*ROCF-DR, No. (%)*							
**Normal**	409 (79.6 %)	216 (80.0 %)	193 (79.1 %)	0.800	187 (80.6 %)	222 (78.7 %)	0.599
**Impaired**	105 (20.4 %)	54 (20.0 %)	51 (20.9 %)		45 (19.4 %)	60 (21.3 %)	
*DSF, No. (%)*							
**Normal**	464 (89.9 %)	244 (90.4 %)	220 (89.4 %)	0.723	216 (92.7 %)	248 (87.6 %)	0.057
**Impaired**	52 (10.1 %)	26 (9.6 %)	26 (10.6 %)		17 (7.3 %)	35 (12.4 %)	
*DSB, No. (%)*							
**Normal**	473 (91.7 %)	249 (92.2 %)	224 (91.1 %)	0.632	209 (89.7 %)	264 (93.3 %)	0.142
**Impaired**	43 (8.3 %)	21 (7.8 %)	22 (8.9 %)		24 (10.3 %)	19 (6.7 %)	
*CSF, No. (%)*							
**Normal**	498 (96.0 %)	259 (95.6 %)	239 (96.4 %)	0.644	231 (98.7 %)	267 (93.7 %)	0.004
**Impaired**	21 (4.0 %)	12 (4.4 %)	9 (3.6 %)		3 (1.3 %)	18 (6.3 %)	
*CSB, No. (%)*							
**Normal**	485 (93.4 %)	258 (95.2 %)	227 (91.5 %)	0.091	226 (96.6 %)	259 (90.9 %)	0.009
**Impaired**	34 (6.6 %)	13 (4.8 %)	21 (8.5 %)		8 (3.4 %)	26 (9.1 %)	
*ROCF-C, No. (%)*							
**Normal**	470 (91.1 %)	255 (94.1 %)	215 (87.8 %)	0.012	209 (89.7 %)	261 (92.2 %)	0.316
**Impaired**	46 (8.9 %)	16 (5.9 %)	30 (12.2 %)		24 (10.3 %)	22 (7.8 %)	
*PVF, No. (%)*							
**Normal**	463 (89.2 %)	243 (89.7 %)	220 (88.7 %)	0.725	208 (88.9 %)	255 (89.5 %)	0.831
**Impaired**	56 (10.8 %)	28 (10.3 %)	28 (11.3 %)		26 (11.1 %)	30 (10.5 %)	
*CVF, No. (%)*							
**Normal**	359 (69.2 %)	179 (66.1 %)	180 (72.6 %)	0.108	159 (67.9 %)	200 (70.2 %)	0.585
**Impaired**	160 (30.8 %)	92 (33.9 %)	68 (27.4 %)		75 (32.1 %)	85 (29.8 %)	
*DS, No. (%)*							
**Normal**	340 (65.8 %)	179 (66.3 %)	161 (65.2 %)	0.790	144 (61.5 %)	196 (69.3 %)	0.066
**Impaired**	177 (34.2 %)	91 (33.7 %)	86 (34.8 %)		90 (38.5 %)	87 (30.7 %)	
ST*, No. (%)*							
**Normal**	483 (94.7 %)	256 (95.2 %)	227 (94.2 %)	0.623	218 (93.2 %)	265 (96.0 %)	0.152
**Impaired**	27 (5.3 %)	13 (4.8 %)	14 (5.8 %)		16 (6.8 %)	11 (4.0 %)	
*ST-E (errors), No. (%)*							
**Normal**	508 (99.6 %)	269 (100.0 %)	239 (99.2 %)	0.134	233 (99.6 %)	275 (99.6 %)	0.907
**Impaired**	2 (0.4 %)	0 (0.0 %)	2 (0.8 %)		1 (0.4 %)	1 (0.4 %)	
*TMTA, No. (%)*							
**Normal**	500 (96.7 %)	263 (97.4 %)	237 (96.0 %)	0.354	226 (96.6 %)	274 (96.8 %)	0.880
**Impaired**	17 (3.3 %)	7 (2.6 %)	10 (4.0 %)		8 (3.4 %)	9 (3.2 %)	
*TMTB, No. (%)*							
**Normal**	498 (99.4 %)	261 (99.6 %)	237 (99.2 %)	0.510	227 (100.0 %)	271 (98.9 %)	0.255
**Impaired**	3 (0.6 %)	1 (0.4 %)	2 (0.8 %)		0 (0.0 %)	3 (1.1 %)	
*MFTC-E (errores), No. (%)*							
**Normal**	511 (98.5 %)	267 (98.5 %)	244 (98.4 %)	0.899	233 (99.6 %)	278 (97.5 %)	0.079
**Impaired**	8 (1.5 %)	4 (1.5 %)	4 (1.6 %)		1 (0.4 %)	7 (2.5 %)	
*MFTC-T (time), No. (%)*							
**Normal**	515 (99.4 %)	269 (99.6 %)	246 (99.2 %)	0.514	234 (100.0 %)	281 (98.9 %)	0.256
**Impaired**	3 (0.6 %)	1 (0.4 %)	2 (0.8 %)		0 (0.0 %)	3 (1.1 %)	
*MFTC-A (accuracy), No. (%)*							
**Normal**	449 (86.5 %)	234 (86.3 %)	215 (86.7 %)	0.908	207 (88.5 %)	242 (84.9 %)	0.239
**Impaired**	70 (13.5 %)	37 (13.7 %)	33 (13.3 %)		27 (11.5 %)	43 (15.1 %)	
*BAI, No. (%)*							
**Normal**	288 (56.1 %)	136 (50.6 %)	152 (62.3 %)	0.007	140 (59.8 %)	148 (53.0 %)	0.123
**Impaired**	225 (43.9 %)	133 (49.4 %)	92 (37.7 %)		94 (40.2 %)	131 (47.0 %)	
*BDI-II, No. (%)*							
**Normal**	276 (53.8 %)	137 (51.1 %)	139 (56.7 %)	0.203	125 (53.4 %)	151 (54.1 %)	0.874
**Impaired**	237 (46.2 %)	131 (48.9 %)	106 (43.3 %)		109 (46.6 %)	128 (45.9 %)	
*BDI-II SA (somatic-affective), No. (%)*							
**Normal**	314 (61.2 %)	159 (59.3 %)	155 (63.3 %)	0.361	141 (60.3 %)	173 (62.0 %)	0.685
**Impaired**	199 (38.8 %)	109 (40.7 %)	90 (36.7 %)		93 (39.7 %)	106 (38.0 %)	
*BDI-II C (cognitive), No. (%)*							
**Normal**	248 (48.3 %)	117 (43.7 %)	131 (53.5 %)	0.026	114 (48.7 %)	134 (48.0 %)	0.876
**Impaired**	265 (51.7 %)	151 (56.3 %)	114 (46.5 %)		120 (51.3 %)	145 (52.0 %)	
*PSQI, No. (%)*							
**Normal**	399 (78.1 %)	221 (83.1 %)	178 (72.7 %)	0.004	204 (88.7 %)	195 (69.4 %)	<0.0001
**Impaired**	112 (21.9 %)	45 (16.9 %)	67 (27.3 %)		26 (11.3 %)	86 (30.6 %)	

Abbreviations: nPH, not hospitalized patient; PH, hospitalized patient; w6m, within 6 months; b6m, beyond 6 months; IADL, instrumental activities daily living scale, IQR, interquartile range, MMSE, mini mental state examination; RAVLT-ST, rey auditory verbal learning test-short term; RAVLT-DR, rey auditory verbal learning test-delayed recall; RAVLT-REC, rey auditory verbal learning test-recognition; ROCF-DR: rey-osterrieth complex figure -delayed recall; DSF, digit span forward; DSB, digit span backward; CSF, corsi span forward; CSB, corsi span backward; ROCF-C, rey-osterrieth complex figure-copy; PVF, phonological verbal fluency; CVF, categorical verbal fluency; DS, WAIS-R digit symbol;ST, stroop test color-word; TMTA, trail making test a; TMTB, trail making test b; MFTC, multiple features target cancellation; BAI, Beck Anxiety Inventory; BDI-II,Beck Depression Inventory; PSQI, Pittsburgh Spleep Quality Index;^§^Number of available tests.

### 5.3 Analysis of impaired neurocognitive test and psychological health according to previous hospitalization for COVID19

According to hospitalization status, we found no significant differences in the Mini-Mental State Examination (MMSE) scores, with 99.6 % of non-hospitalized patients and 98 % of previously hospitalized patients showing normal results (p = 0.108). However, a higher proportion of previously hospitalized patients exhibited impairments in the Rey Auditory Verbal Learning Test for Short-Term Recall (RAVLT-ST) and Long-Term Recognition (RAVLT-REC), with 14.5 % vs. 8.9 % (p = 0.044) and 24.5 % vs. 15.1 % (p = 0.007) by comparing previously hospitalized vs. not, respectively, reflecting deficits in short-term and long-term verbal episodic memory.

Additionally, the Rey-Osterrieth Complex Figure – Copy (ROCF-C) test was more frequently impaired in previously hospitalized patients than in not previously hospitalized patients (12.2 % vs. 5.9 %; p = 0.012), suggesting compromised constructional praxis. However, the Instrumental Activities of Daily Living (IADL) assessment showed no significant differences in autonomy for instrumental daily life functions between the two groups (Table 2, left panel).

Regarding psychological health, the Beck Anxiety Inventory (BAI) score >85 % was observed more frequently in not previously hospitalized patients compared to previously hospitalized patients (49.4 % vs. 37.7 %; p = 0.007), while the Beck Depression Inventory-II (BDI-II) score >85 % was seen in 48.9 % vs. 43.3 % (p = 0.203). Impaired cognitive performance was noted in 56.3 % vs. 46.5 % of PH patients (p = 0.026), and sleep disturbances (PSQI >5) in 27.3 % vs. 16.9 % (p = 0.004) (Table 2, left panel).

The relationship between the cognitive outcome (CO), defined as having at least one impaired test in the neuropsychological assessment (NPA), and psychological health and sleep quality showed significant associations with both Beck Anxiety Inventory >85 % and Beck Depression Inventory-II >85 % (97.3 % vs. 82.29 %, p < 0.001 and 100 % vs. 79.3 %, p < 0.001, respectively). However, no significant association was found with Pittsburgh Sleep Quality Index >5 (91.1 % vs. 87.9 %, p = 0.361).

### 5.4 Relationships of CO and psychological symptoms

We found a significantly higher proportion of patients with impaired performance in several cognitive domains among those with higher Beck Anxiety Inventory (BAI) scores (>85 %). Specifically, these patients showed deficits in verbal short-term learning (RAVLT-ST: 15.2 % vs. 8.8 %, p = 0.025), long-term memory (RAVLT-DR: 12.6 % vs. 5.8 %, p = 0.006), short-term and working memory capacity for verbal (DSF, DSB) and visuospatial stimuli (CSF, CSB) (DSF: 16.1 % vs. 5.1 %, p < 0.001; DSB: 11.7 % vs. 5.8 %, p = 0.016; CSF: 5.6 % vs. 2.0 %, p = 0.029; CSB: 9.5 % vs. 4.1 %, p = 0.012), as well as slower psychomotor processing speed (DS: 16.1 % vs. 5.1 %, p < 0.001; TMTA: 9.5 % vs. 4.1 %, p = 0.012).

Similarly, among those with higher Beck Depression Inventory-II (BDI-II) scores (>85 %), we observed impairments in verbal long-term memory (RAVLT-DR: 13.5 % vs. 5.0 %, p < 0.001), recognition of verbal stimuli in long-term memory (RAVLT-REC: 23.8 % vs. 15.4 %, p = 0.016), short-term memory for visuospatial stimuli (CSF: 9.0 % vs. 4.3 %, p = 0.029), and psychomotor processing speed (DS: 16.1 % vs. 5.1 %, p < 0.001; TMTA: 4.9 % vs. 1.4 %, p = 0.021).

Finally, patients with higher Pittsburgh Sleep Quality Index (PSQI) scores (>5) demonstrated impairments in short-term memory capacity for verbal (DSF: 15.7 % vs. 8.6 %, p = 0.028) and visuospatial stimuli (CSF: 15.2 % vs. 8.5 %, p = 0.001), as well as working memory for visuospatial stimuli (CSB: 11.0 % vs. 5.2 %, p = 0.023).

### 5.5 Factors associated with psychological symptoms

In univariate crude odd ratios (ORs) analysis for psychological symptoms, female gender resulted a risk factor of altered scores in Beck Anxiety Inventory>85 % [OR 1.81, 95 % CI 1.27 to 2.58, p = 0.001], Beck Depression Inventory-II >85 % [OR 1.43, 95 % CI 1.01 to 2.04, p = 0.042] and Pittsburgh Sleep Quality Index >5 [OR 2.27, 95 % CI 1.46 to 3.55, p < 0.001], suggesting that women are more likely to shown anxious-depressive and sleep disorders, respectively.

Female gender is also associated with a higher risk of impaired performance in cognitive test, such as Multiple Features Target Cancellation [OR 1.79, 95 % CI 1.05 to 3.03, p = 0.030], suggesting a lower accuracy in the visual search of multiple targets in a restricted time; whereas, this variable become protective towards Rey Osterrieth Complex Figure-Delayed Recall (visual memory of a complex geometric figure) [OR 0.60, 95 % CI 0.39 to 0.93, p = 0.023].

Particularly, previous hospitalization for COVID19 seems to be a risk factor of impaired Rey Osterrieth Complex Figure*-* Copy test, suggesting a reduced ability of constructional praxis [OR 2.22, 95 % 1.84 to 4.18, p = 0.013], in Beck Anxiety Inventory >85 % (related to anxious symptoms) [OR 0.61, 95 % 0.43 to 0.88, p = 0.008] and in Pittsburgh Sleep Quality Index >5 [OR 1.84, 95 % CI 1.20 to 2.83, p = 0.005] (presence of sleep disorders).

For participants with NPA for more than 6 months, scores in Pittsburgh Sleep Quality Index >5 (sleep quality) and Corsi Span Forward (visual-spatial memory) appear to be impaired [OR 3.46, 95 % 2.13 to 5.59, p < 0.0001 and 5.59, 95 % 1.50 to 17.84, p = 0.009; respectively].

Moreover, having at least 1 comorbidity seems to be associated with pathological scores in Beck Depression Inventory-II (depressive symptoms) [OR 1.55, 95 % 1.08 to 2.24, p = 0.017] and with Rey Osterrieth Complex Figure- Copy (constructional praxis) [OR 2.39, 95 % CI 1.30 to 4.41, p = 0.005].

Finally, Rey Auditory Verbal Learning Test for long-term memory - Recognition*,* used to describe verbal episodic memory in terms of relief from a facilitating condition, seems to be altered in women, in people having at least 1 comorbidity and in PH [OR 0.54, 95 % CI 0.35 to 0.84, p = 0.007; OR 2.47, 95 % CI 1.59 to 3.85, p=<0.001 and OR 1.81, 95 % CI 1.16–2.83, p = 0.008, respectively].

The last interesting data is about Categorical Verbal Fluency test, used for characterizing categorical verbal fluency, which seems to be influenced by treatment with remdesivir [OR 0.53, 95 % CI 0.30–0.95, p = 0.033].

**Fig. 2:**
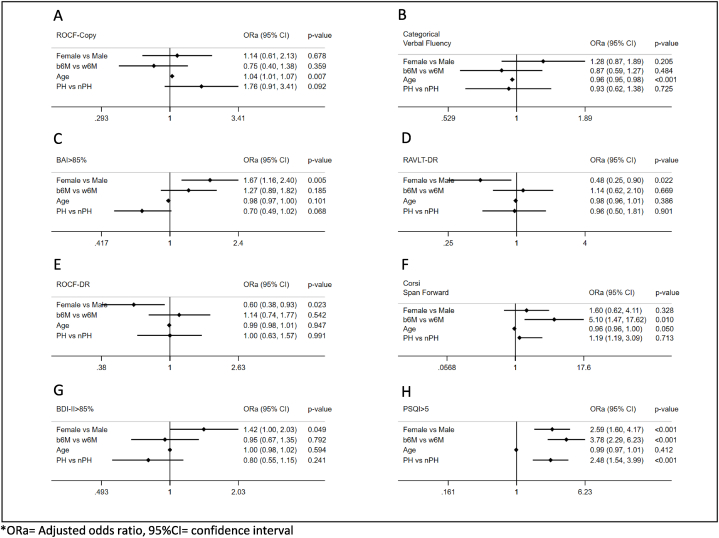
Multivariable logistic regression models for neuropsychological alterations: ROCF-Copy (A); Categorical Verbal Fluency (B); BAI>85 % (C); RAVLT-DR (D); ROCF-DR(E); Corsi Span Forward (F); BDI-II>85 % (G) and PSQI>5 (H).

In the multivariable logistics regression models, the risk of impairment in Rey Auditory Verbal Learning Test for short term-memory*-*Delayed Recall and Rey Osterrieth Complex Figure- Delayed Recall, (altered performance in verbal long-term memory and long-term memory for visual-spatial stimuli) decrements in male gender [OR 0.48, 95 % 0.26 to 0.90, p = 0.022 and OR 0.60, 95 % CI 0.39 to 0.93, p = 0.023; respectively]. Marginally, the risk of impairment in Rey Osterrieth Complex Figure- Copy was associated with age [OR 1.04, 95 % CI 1.01 to 1.07, p = 0.007], as well as Categorical Verbal Fluency [per 1-year increase OR 0.96, 95 % CI 0.95–0.98, p < 0.001] (altered performance in constructional praxis and categorical fluency and language programming). Furthermore, being evaluated b6m is associated with a higher risk of impairment in Corsi Span Forward [OR 5.10, 95 % CI 1.47–17.62, p = 0.010] (short-term memory for visuo-spatial stimuli) (A). Moreover, the probability of obtaining pathological scores in Beck Anxiety Inventory>85 % and Beck Depression Inventory-II>85 % increases for female gender [OR 1.67, 95 % CI 1.16–2.40, p = 0.005 and OR 1.42, 95 % CI 1.00 to 2.03, p = 0.049], as well as Pittsburgh Sleep Quality Index>5 increases for female gender [OR 2.59, 95 % CI 1.61 to 4.17, p < 0.001], for evaluations beyond 6 months [OR 3.78.95 % CI 2.29 to 6.22, p < 0.001] and hospitalized patients [OR 2.49, 95 %, 95 % CI 1.54 to 3.99, p < 0.001] (Fig. 2).

## 6 Discussion

Our research provides an important insight into the cognitive issues associated with Post-Acute COVID-19 Syndrome (PACC), emphasizing the significant role of neuropsychological assessments in identifying subtle cognitive deficits that patients may not self-report. The findings highlight several key aspects. First, the importance of neuropsychological assessments for PACC patients, the specific tests used are crucial, and our study suggests that certain tests are more sensitive in detecting subtle cognitive difficulties that patients might not even notice [24]. Second, certain cognitive functions, such as working memory, visual-spatial memory, and processing speed, are particularly impaired six months after COVID-19 infection.

A large proportion of patients, both symptomatic and asymptomatic, displayed cognitive impairments, particularly in areas like working memory, visual-spatial memory, and processing speed. The study found that these impairments were persistent even six months after the initial infection, with processing speed being one of the most affected functions. This is consistent with other studies that also reported post-hospitalization impacts on cognitive functions, including verbal short-term learning (RAVLT-ST), long-term verbal memory (RAVLT-DR), and constructional praxis (ROCF-C) [25,26]. The detailed neuropsychological assessments used in this study allowed for the identification of more subtle impairments, which might have been missed with less rigorous testing methods like the Mini Mental State Examination (MMSE).

Third, the study suggests a strong link between cognitive impairments and psychological issues such as anxiety and depression. Anxiety and depression were found to be more prevalent among patients with cognitive complaints, echoing findings from other research. This highlights the complex interplay between psychological health and cognitive function in PACC patients, where issues like fatigue and sleep disturbances may exacerbate cognitive difficulties [[27], [28], [29]].

Finally, women seem to be at higher risk for both cognitive and psychological problems post-COVID. This is consistent with other studies suggesting that women are more likely to report symptoms of long COVID and seek medical assistance. Female gender was associated with a higher likelihood of anxiety-depressive illnesses and sleep disorders, which may further compound cognitive issues [30,31].

The study has several limitations, including the absence of data on SARS-CoV-2 vaccinations, virus variants, or pre-COVID-19 cognitive baselines. These factors, along with the lack of a healthy control group, restrict the ability to draw definitive conclusions about the cognitive effects of COVID-19. Additionally, while our study provides valuable insights, population-based epidemiological studies are necessary to determine the exact prevalence of cognitive impairments in PACC patients.

Despite these limitations, the study has notable strengths. The large sample size and the use of a standardized, comprehensive battery of cognitive tests enhance the accuracy of the findings. The adjustment of test scores based on age and education variables further increases the precision of the cognitive assessments. Furthermore, the inclusion of psychological and sleep assessments adds depth to the understanding of the broader impacts of COVID-19 on patients' cognitive and mental health.

In conclusion, our research underscores the importance of thorough neuropsychological evaluations in identifying cognitive impairments in PACC patients. The connections between cognitive decline, psychological distress, and gender differences add crucial insights to the understanding of long-term COVID-19 impacts. Future studies could benefit from including healthy control groups, data on vaccination status, and further exploration of language difficulties, which remain underexplored in current research.

## 7 Conclusions

Our study on PACC shows a correlation between psychological and cognitive symptoms. COVID-19 can cause persistent neurocognitive outcomes, especially in executive functions, attention, and working memory. We recommend comprehensive neurocognitive assessments for all PACC patients, regardless of reported symptoms, and suggest specialized clinics and support groups to address this burden. Further research is needed to understand the extent of cognitive dysfunction and its socio-economic implications, urging governments and health systems to prioritize care for PACC patients.

## CRediT authorship contribution statement

**Alessandra Vergori:** Writing – review & editing, Writing – original draft, Visualization, Project administration. **Giulia Del Duca:** Writing – review & editing, Writing – original draft, Visualization, Validation, Project administration, Methodology, Investigation, Data curation. **Paola Borrelli:** Writing – review & editing, Writing – original draft, Formal analysis. **Anna Clelia Brita:** Writing – original draft, Methodology, Data curation. **Carmela Pinnetti:** Writing – original draft, Visualization, Conceptualization. **Ilaria Mastrorosa:** Visualization, Investigation. **Marta Camici:** Visualization, Investigation. **Annalisa Mondi:** Visualization, Investigation. **Valentina Mazzotta:** Visualization, Investigation. **Pierangelo Chinello:** Visualization, Investigation. **Paola Mencarini:** Visualization, Investigation. **Maria Letizia Giancola:** Visualization, Investigation. **Amina Abdeddaim:** Visualization, Investigation. **Enrico Girardi:** Supervision. **Andrea Antinori:** Supervision.

## Data sharing statement

All data will be available upon reasonable request to the corresponding author.

## Funding

The NeuroCOVID study was supported by funding received from Italian Ministry of Health: Ricerca Corrente Linea 1.

## Declaration of competing interest

The authors declare that they have no known competing financial interests or personal relationships that could have appeared to influence the work reported in this paper.
