# Supplementary material for: Cognitive outcomes and psychological symptoms in an Italian cohort with post-acute COVID-19 condition (PACC)
Source: Heliyon. 2024 Oct 16;10(20):e39431. doi: 10.1016/j.heliyon.2024.e39431 (PMC11513557; doi:10.1016/j.heliyon.2024.e39431)
Supplement: Multimedia component 1 [file mmc1.docx]

**Appendix A. Supplementary material**

*Table of contents*

- Study procedures………………………..………………………………………...page 2
- References…………………………………………………………………………page 4,5
- Study protocol Version 1.0 10.05.2020……………………………………………page 5-19
- Study protocol references…………………….………………………………........page 19-20

**Study Procedures**

*Neuropsychological assessment (NPA)*

The neurocognitive tests used were: Mini Mental State Examination (MMSE) to assess global cognitive status, Rey Auditory Verbal Learning Test-Short Term (RAVLT-ST) to assess verbal short-term learning , Rey Auditory Verbal Learning Test-Delayed Recall (RAVLT-DR) and Rey Auditory Verbal Learning Test-Recognition (RAVLT-REC) to assess verbal long-term memory and recognition of verbal stimuli in long-term memory; Digit Span Forward (DSF) and Backward (DSB) to assess short-term and working memory capacity for verbal stimuli; Corsi Span Forward (CSF) and Backward (CSB) to assess short-term and working memory capacity for visuo-spatial stimuli; Rey-Osterrieth Complex Figure- Copy (ROCF-C) to assess constructional praxis; Rey-Osterrieth Complex Figure -Delayed Recall (ROCF-DR) to assess long-term memory for visual-spatial stimuli; Trail Making Test A (TMTA) and WAIS-R Digit Symbol (DS)to assess speed of psycho-motor processing; Trail Making Test B (TMTB) to assess shifting ability and Stroop Test Color-Word (ST) to assess inhibition an automatic reply and mental flexibility; Multiple Features Target Cancellation (MFTC) to assess attention and Phonological and Categorical Verbal Fluency (living and non-living, PVF and CVF) to assess fluency and language programming. We use the Instrumental activities daily living scale (IADL) to assess the degree of autonomy in instrumental activities of daily living.

In addition, the Beck Anxiety Inventory (BAI) which assess the cognitive and physiological symptoms of anxiety, the Beck Depression Inventory (BDI-II), which assess cognitive, affective, and physiological symptoms of depression (Somatic-affective SA/ Cognitive C dimensions) were administered to evaluate the presence of anxious and depressive symptoms (scores >85% indicates the presence of pathological symptoms), and the Pittsburgh Sleep Quality Index (PSQI) for sleep quality assessment (if score >5 indicates the presence of poor sleep quality).

The neurocognitive tests used were:

*Mini Mental State Examination* (MMSE) [1]

*Rey Auditory Verbal Learning Test*-*Short Term* (RAVLT-ST), *Rey Auditory Verbal Learning Test*-*Delayed Recall* (RAVLT-DR), *Rey Auditory Verbal Learning Test*-*Recognition* (RAVLT-REC) [2]

*Digit Span Forward* (DSF) and *Backward* (DSB), *Corsi Span Forward* (CSF) and *Backward* (CSB) [3]

*Rey-Osterrieth Complex Figure- Copy* (ROCF-C), *Rey-Osterrieth Complex Figure* -*Delayed Recall* (ROCF-DR) [4]

*Trail Making Test A* (TMTA) [5] *Trail Making Test B* (TMTB) [5]

*WAIS-R Digit Symbol* (DS) [6]

*Stroop Test Color-Word* (ST) [7]

M*ultiple Features Target Cancellation* (MFTC) [8]

*Phonological and Categorical Verbal Fluency* (living and non-living, PVF and CVF) [9] *Instrumental activities daily living scale* (IADL) [10]

*Beck Anxiety Inventory* (BAI) [11]

*Beck Depression Inventory* (BDI-II) [12]

*Pittsburgh Sleep Quality Index* (PSQI) [13]

PACC-CO was classified based on the scores into equivalent scores (ES) in each test and defined by the presence of alterations (<cutoff-ES = 0) in at least one test [14]. ES makes it possible to establish the test subject's position relative to normal subjects, net of the influence of variables related to gender, age and education. Depending on the test and the basic demographic variables (gender, age and education), the raw score obtained by the test subject is adjusted and then converted into the corresponding ES, classified on a 5-level scale: ES= 0 Deficient comprising; ES = 1 Borderline performance; ES = 2-3 Middle-lower comprising performance; ES = 4 Middle-upper comprising performance. The presence of PACC-CO was assessed, cognitive domains and tests deficits were classified, and diagnostic criteria were established for clinical definition in line with currently available references in the DSM-5 [15].

**References**

1. Measso G, et al. The Mini-Mental State Examination: normative study of an italian random sample. Developmental Neuropsycol 1993; 9:77-85. DOI: 10.1111/j.1468-1331.1996.tb00423.x
2. Carlesimo GA, Caltagirone C, Gainotti G. The mental deterioration battery: normative data diagnostic reliability and qualitative analyses of cognitive impariment. The group for the standardization of the mental deterioration battery. Eur Neurol. 1996;36(6):378-84. DOI: 10.1159/000117297
3. Monaco M, et al. Forward and backward span for verbal and visuo-spatial data: standardization and normative data from an italian adult population. Neurol Sci. 212 Jun12. DOI: 10.1007/s10072-012-1130-x
4. Caffarra P, et al. Rey-Osterrieth complex figure: normative values in an italian population sample. Neurol sci. 2002 Mar; 22(6):443-7. DOI: 10.1007/s100720200003
5. Giovagnoli AR, et al. Trail making test: normative values from 287 normal adult controls. Ital J Neurol Sci. 1996 Aug; 17(4):305. DOI: 10.1007/BF01997792
6. Orsini A, Laicardi C. WAIS-R. Contributo Alla Taratura Italiana. Firenze: Giunti O.S. Organizzazioni Speciali,1997.
7. Caffarra P, et al. Una versione abbreviata del test di Stroop: dati normativi nella popolazione italiana. Rivista di neurologia 2002; 12: n4.
8. Marra C, et al. The multiple features target cancellation (MFTC): an attenzional visual conjunction search test. Normative values for the italian population. Neurol Sci. 2012 Feb 19 DOI: 10.1007/s10072-012-0975-3
9. Marra C, Ferraccioli M, Gainotti G. Gender-related dissociations of categorical fluency in normal subjects and in subjects with Alzheimer’s disease. Neuropsychology. 2007 Mar; 21(2):207-11 DOI: 10.1037/0894-4105.21.2.207
10. Lawton MP, Brody EM. Assessment of older people: self-maintaining and instrumental activities of daily living. Gerontologist. 1969 Autumn;9(3):179-86. PMID: 5349366.
11. Beck, A. T., & Steer, R. A. Manual for the Beck anxiety inventory. San Antonio, TX: Psychological Corporation 1990.
12. Beck A., Steer R., & Brown G. Beck Depression Inventory. 2nd edition. San Antonio, Tex, USA: The Psychological Corporation 1996.
13. Curcio G, Tempesta D, Scarlata S, et al. Validity of the Italian version of the Pittsburgh Sleep Quality Index (PSQI). Neurol Sci. 2013;34(4):511-51 DOI: 10.1007/s10072-012-1085-y
14. Capitani, E. et Laiacona, M. Composite neuropsychological batteries and demographic correction: standardization based on equivalent scores, with a review of published data. The Italian Group for the Neuropsychological Study of Ageing. Journal of Clinical and Experimental Neuropsychology Vol. 19, 1997, No. 6, pp. 795-809. DOI: 10.1080/01688639708403761
15. Anastasi, A. Diagnostic and Statistical Manual of Mental Disorders, Fifth Edition, DSM-5. Raffaello Cortina Editore, Milano, 2014.

**Study protocol
Version 1.0- 10.05.2020**

**Analisi delle manifestazioni neurologiche e dei disturbi neurocognitivi in corso di infezione da nuovo coronavirus 2019 (SARSCOV2). Studio NeuroCovid.**

| **Responsabile dello studio** | Dott. Andrea Antinori |
| --- | --- |
| **Affiliazione e contatti** | Direttore del Reparto di Immunodeficienze Virali del Dipartimento Clinico  INMI “L. Spallanzani”  Via Portuense, 292  00149 ROMA  Tel. +39-0655170546  Fax +39 0655170477  e-mail [andrea.antinori@inmi.it](mailto:andrea.antinori@inmi.it) |
| **Strutture coinvolte** | **Dipartimento Clinico:**  Dott.ssa Carmela Pinnetti, Dott.ssa Alessandra Vergori, Dott. Andrea Antinori  **Laboratorio di Immunologia Cellulare:**  Dott.ssa Chiara Agrati, Dott.ssa Veronica Bordoni, Dott.ssa Stefania Notari, Dott. Massimo Tempestilli  **Laboratorio di Terapia Monitoraggio Farmaci Antiretrovirali:**  Dott.ssa Federica Forbici, Prof Carlo Federico Perno  **Laboratorio di Virologia:**  Dott.ssa Concetta Castilletti, Dott.ssa Maria Rosaria Capobianchi  **Epidemiologia**:  Dott. Enrico Girardi, Dott. Pierluca Piselli  Istituto di Radiologia  Prof. Paolo Campioni, Dott. Vincenzo Schininà |
| **Tipologia di studio** | Monocentrico, osservazionale prospettico |
| **Versione** Proto**collo**   \|  \| \| --- \| | Versione 1- 10.05.2020 |

**INDICE**

| **PREMESSA E RAZIONALE………………………………………….................................** | **4** |
| --- | --- |
| **OBIETTIVI DELLO STUDIO……………………………………………….......................** | **5** |
| **DISEGNO DELLO STUDIO…………………………:::…………………………………..** | **6** |
| **DISEGNO SPERIMENTALE::…………………………………………………………….** | **6** |
| **ARRUOLAMENTO DEI PAZIENTI………………………………………………………** | **7** |
| **DIMENSIONE DEL CAMPIONE……..…………………………………………………..** | **8** |
| **DURATA DELL’ARRUOLAMENTO DEI PAZIENTI.....................................................** | **9** |
| **TRATTAMENTI FARMACOLOGICI NELL’AMBITO DEL STUDIO ………………** | **9** |
| **DATABASE** ……………………...…………………............................................................... | **9** |
| ASSESSMENT NEUROCOGNITIVO ……………….……………………............................ | **9** |
| **PROCEDURE DI LABORATORIO …………………………………..…………………...** | **14** |
| **PROCEDURE DI NEUROIMAGING……..……………….………………………………** | **15** |
| **ASPETTI ETICI E TUTELA DELLA PRIVACY……………….…………………………** | **16** |
| **FONTE DEI FINANZIAMENTI……….……………………………………………………** | **17** |
| **RICADUTA SUL SISTEMA SANITARIO NAZIONALE………………………………...** | **17** |
| **BIBLIOGRAFIA………………………………………..........................................................** | **17** |

**PREMESSA E RAZIONALE**

La malattia da nuovo coronavirus 2019 (COVID-19) è un'infezione emergente del tratto respiratorio causata da un coronavirus, SARS-CoV-2, descritto per la prima volta a Wuhan, in Cina, nel dicembre 2019. Sebbene la maggior parte delle persone con COVID-19 sviluppi malattie lievi o non complicate, tuttavia nel circa 15% dei casi è descritto un decorso grave che richiede ospedalizzazione e supporto respiratorio e nel 5-10% dei pazienti ammissione in un'unità di terapia intensiva (UTI).

Tale epidemia, sta coinvolgendo tutti i Paesi del mondo con e i dati sono in costante aumento: in base ai dati forniti giornalmente dall’ ECDC (Agenzia Europea perla Prevenzione ed il Controllo delle Malattie), integrati con quelli che per l’Italia fornisce la Protezione Civile Nazionale, ad oggi (07 maggio 2020) i casi accertati complessivi sono 3716319, con 263775 decessi [1, 2]. Ad oggi sono complessivamente 205 le nazioni e i territori con almeno un caso di positività [3]. E’ noto che gli agenti virali a RNA possano colpire organi e sistemi extra-respiratori, incluso il Sistema Nervoso (SN) con meccanismi di coinvolgimento non ancora definiti. Specificamente per quanto riguarda il SN, la neuro invasione potrebbe avvenire per via ematogena o per trasporto assonale retrogrado attraverso alcuni nervi cranici (fra cui l’olfattorio, il trigemino, il nervo glossofaringeo e il vago) o nervi periferici [4]. La pandemia di recente insorgenza causata da SARS-CoV-19 ha riacceso l’attenzione sul possibile neurotropismo di questo virus e quindi il possibile interessamento del Sistema Nervoso centrale (SNC) e periferico (SNP). Complicanze psichiatriche e neurologiche erano state riportate già durante l’epidemia di SARS nel2003[5].A parte le alterazioni dell’umore in senso depressivo, disturbo d’ansia e idee suicidarie, sono stati segnalati casi di allucinosi organica(allucinazioni visive e uditive), disturbi comportamentali, deliri di persecuzione, disorientamento temporo-spaziale, disturbo ipomaniacale[5]. Altre segnalazioni hanno riportato casi isolati di encefalomielite fatale da Coronavirus OC43 a fronte di lieve interessamento polmonare[4]e crisi comiziali generalizzate in pazienti con infezione e positività liquorale per SARS-CoV [6].

Recenti dati suggeriscono che anche il SARS-CoV-2 raggiunga il sistema nervoso centrale [7] mediante il legame con il suo noto recettore ACE-2 espresso dalle cellule gliali e neuronali che ne permetterebbe il passaggio attraverso la lamina cribrosa dell’etmoide in prossimità del nervo olfattorio. A supporto di ciò, un’analisi multicentrica ha dimostrato che anosmia e ageusia sono disordini presenti frequentemente tra le manifestazioni cliniche di malattia da SARS-CoV-2, suggerendo una via di infezione nasale con un possibile accesso diretto al SN [8] Il potenziale ruolo della barriera ematoencefalica (BEE) nel contenere e nel prevenire l’accesso al tessuto cerebrale è, tuttavia ancora da chiarire. La recente segnalazione della perdita del processo involontario del respiro come anche le manifestazioni neurologiche riportate nel 36% dei casi analizzati dalla casistica cinese (tra cui patologie cerebrovascolari acute, alterazione dello stato di coscienza, sintomi muscolo scheletrici con incremento delle CPK)[9] hanno riacceso l’interesse sul coinvolgimento del SN in corso di infezione da COVID-19 e sui meccanismi patogenetici ad essa correlati [10]. Di recente sono stati documentati quadri di encefalopatia emorragica necrotizzante (forma rara osservata in passato nel corso di altre infezioni virali, fra cui l’influenza) legati all’infezione da SARS-CoV-2, posti in relazione alla marcata risposta infiammatoria, anche intracranica associata a encefalite da coronavirus [11,12]. La sintomatologia neurologica si può manifestare con uno spettro di segni e sintomi molto eterogeneo e, in alcuni casi, sovrapposto a patologie pregresse del SN a genesi multifattoriale, tra cui ictus nel 6%dei casi (il virus influenza profondamente i meccanismi della coagulazione), alterazioni dello stato di coscienza (confusione, stato soporoso, ecc) nel 15% e come danno muscolare nel 19%, acroparestesie e sintomi da encefalite [12, 13]. Le evidenze in merito alla *detection* virale mediante RT PCR su liquor sono, al momento molto esigue ed il danno neurologico può essere legato alla risposta infiammatoria causata dal virus stesso. Alla luce di questi dati si può quindi supporre che l’interessamento del Sistema Nervoso sia Centrale che Periferico sia presente in un certo numero dei pazienti con infezione da SARS-CoV-2.

**OBIETTIVI DELLO STUDIO**

Obiettivi del nostro Studio sono:

- valutare la presenza di segni e sintomi neurologici in pazienti con infezione da SARS-CoV-2 e caratterizzarne la loro evoluzione nel tempo;

- valutazione dell’*assessment* neuro cognitivo dei pazienti mediante esecuzione di una batteria completa di test che esplorino i cinque domini esplorati;

- descrizione dei quadri di *neuroimaging* mendiante esecuzione di TAC con studio perfusionale e/o RMN encefalo con studio funzionale;

- caratterizzazione virologica ed immunologica su campioni di sangue periferico e liquorale (ove possibile eseguire una puntura lombare diagnostica) e del danno di barriera, mediante:

1. Valutazione dell’attivazione immunitaria e della presenza di fattori proinfiammatori, effettuata misurando biomarcatori di infiammazione ed immunoattivazione (fra cui neopterina, sCD14, MCP-1, IL-6, IL-8, IP-10, MCP-1 and G-CSF) e di danno neuronale (NF-L ed altri) sia nel compartimento neuronale che in quello plasmatico.
2. Caratterizzazione dei linfociti T, B e NK nel sangue periferico e nel CSF (attivazione, maturazione, espressione di catene kappa e lambda) e loro associazione con i marcatori infiammatori e attivatori e con le caratteristiche cliniche.
3. Determinazione del danno di barriera ematoencefalica (mediante la misurazione dei livelli di albumina sia a livello plasmatico che liquorale ed il loro rapporto, ovvero mediante la misurazione dei livelli di IgG).
4. Valutazione delle differenze virologiche in coppie liquor/plasma, attraverso l'analisi comparativa della carica virale, dei sottotipi virali presenti nei due comparti ove possibile ottenere tale dato, della risposta anticorpale nel compartimento plasmatico ed intratecale.

**DISEGNO DELLO STUDIO**

Studio monocentrico retrospettivo e prospettico.

**DISEGNO SPERIMENTALE**

Arruolamento di pazienti con documentata infezione da SARS-CoV-2 (presenza di SARS-CoV-2 RNA su campione biologico ovvero sierologia positiva per SARS-CoV-2 e quadro clinico/polmonare compatibile) e segni/sintomi neurologici, seguiti consecutivamente presso il Dipartimento Clinico dell’Istituto Nazionale per le Malattie Infettive “L. Spallanzani”, Roma. I pazienti, ovvero i loro rappresentanti legali, dovranno sottoscrivere consenso informato per la partecipazione allo studio. Saranno raccolti retrospettivamente dall’analisi delle cartelle cliniche i principali dati riguardanti esami ematochimici e radiologici, sintomatologia e segni neurologici, pregressi patologici, terapia effettuata. Sarà compilato un file anonimizzato in cui verranno inserite tali informazioni e il *follow up* dei pazienti. I pazienti effettueranno laddove è possibile uno screening neurocognitivo, effettueranno studio di *imaging* e se non controindicato verrà proposta esecuzione di puntura lombare diagnostica. Tali esami ove richiesto dalla situazione clinica del paziente e su giudizio del medico curante potranno essere ripetuti dopo adeguata terapia a distanza di 1-3 mesi.

**ARRUOLAMENTO DEI PAZIENTI**

Saranno considerati includibili nello studio, dati estrapolati dalla cartella clinica di pazienti seguiti e campioni stoccati di plasma disponibili e non più utilizzabili per ulteriori finalità cliniche da pazienti con età > di 18 anni:

- **Utili per lo Studio Retrospettivo**

1. Al momento ancora seguiti ed in follow up attivo presso le Strutture dell’Istituto Nazionale per le Malattie Infettive “L. Spallanzani”. Resta fermo l'obbligo di raccogliere il consenso al trattamento dei dati degli interessati inclusi nella ricerca in tutti i casi in cui, nel corso dello studio, sia possibile rendere loro un'adeguata informativa e, in particolare, laddove questi si rivolgano al centro di cura, anche per visite di controllo sarà richiesta sottoscrizione di Consenso Informato (ALLEGATO A).
2. Al momento non più seguiti e non in follow up attivo presso le Strutture dell’Istituto Nazionale per le Malattie Infettive “L. Spallanzani”. (Nel qual caso si verifica la situazione di impossibilità di fornire l’informativa agli interessati). E’ una situazione del tutto particolare o eccezionale, che riguarda solo una parte dei dati e dei campioni residui che, tuttavia, è indispensabile includere nell’analisi per le seguenti motivazioni:

a. Motivi etici riconducibili alla circostanza che l'interessato ignora la propria condizione. In questa categoria possiamo includerei campioni utilizzati per le ricerche per le quali l'informativa sul trattamento dei dati da rendere agli interessati comporterebbe la rivelazione di notizie concernenti la conduzione dello studio la cui conoscenza potrebbe arrecare un danno materiale o psicologico agli interessati stessi (ad esempio, gli studi epidemiologici sulla distribuzione di un fattore che predica o possa predire lo sviluppo di uno stato morboso per il quale non esista un trattamento).

b. Motivi di impossibilità organizzativa riconducibili alla circostanza che la mancata considerazione dei dati riferiti al numero stimato di interessati che non è possibile contattare per informarli, rispetto al numero complessivo dei soggetti che si intende coinvolgere nella ricerca, produrrebbe conseguenze significative per lo studio in termini di alterazione dei relativi risultati; ciò avuto riguardo, in particolare, ai criteri di inclusione previsti dallo studio, alle modalità di arruolamento, alla numerosità statistica del campione prescelto, nonché al periodo di tempo trascorso dal momento in cui i dati riferiti agli interessati sono stati originariamente raccolti (i campioni potrebbero essere inclusi nell’analisi di studi riguardo interessati con patologie ad elevata incidenza di mortalità o in fase terminale della malattia o in età avanzata e in gravi condizioni di salute). Con riferimento a tali motivi di impossibilità organizzativa, è autorizzato il trattamento dei dati di coloro i quali, all'esito di ogni ragionevole sforzo compiuto per contattarli, anche attraverso la verifica dello stato in vita, la consultazione dei dati riportati nella documentazione clinica, l'impiego dei recapiti telefonici eventualmente forniti, nonché l'acquisizione dei dati di contatto presso l'anagrafe degli assistiti o della popolazione residente, risultino essere al momento dell'arruolamento nello studio:

- deceduti

- non contattabili

- **Utili per lo Studio Prospettico**

1. Pazienti a cui verrà proposta esecuzione di esame di *neuroimaging* previa sottoscrizione di Consenso Informato all’esame su modulo aziendale, e previa sottoscrizione di Consenso Informato dello Studio (ALLEGATO A)
2. Pazienti a cui verrà proposta puntura lombare diagnostica per esigenze cliniche, previa sottoscrizione di Consenso Informato alla manovra su modulo aziendale, e previa sottoscrizione di Consenso Informato dello Studio (ALLEGATO A)

A tale proposito appare utile sottolineare due aspetti di importanza fondamentale nell’ambito dell’analisi dei campioni utilizzabili per tale protocollo:

- I campioni analizzabili sono campioni residuali non altrimenti utilizzabili per ulteriori finalità cliniche ottenuti da manovra invasiva effettuata per finalità cliniche, la cui ripetizione è stata o è proposta ed eseguita per ragioni strettamente diagnostiche rispetto alla problematica medica del paziente, non con la finalità specifica di raccolta di tali campioni.
- Su tali campioni saranno effettuati esami virologici ed immunologici non validati al momento attuale nella pratica clinica routinaria e che, pertanto, non avrebbero potuto o potrebbero conferire immediato vantaggio clinico per il singolo paziente, ma la cui analisi nell’ambito di una coorte di pazienti molto ampia potrebbe permettere di spiegare meccanismi patogenetici non altrimenti caratterizzabili di danno neuronale.

**DIMENSIONE DEL CAMPIONE**

E’ previsto l’arruolamento di un numero minimo di 50 pazienti. In un sottogruppo di 10 pazienti verrà eseguita puntura lombare diagnostica.

**DURATA DELL’ARRUOLAMENTO DEI PAZIENTI**

Lo studio prevede l’analisi retrospettiva di campioni già disponibili e stoccati presso la Banca Biologica di pazienti con patologia neurologica documentata clinicamente. L’analisi prospettica durerà almeno sei mesi. In base ai risultati ottenuti, potrà eventualmente essere inoltrata la richiesta di estensione del periodo di studio.

**TRATTAMENTI FARMACOLOGICI NELL’AMBITO DELLO STUDIO**

La terapia antivirale e ogni altro tipo di terapia farmacologica nell’ambito del Progetto verrà prescritta sulla base delle linee-guida vigenti, le necessità del paziente e l’esperienza del Medico Curante. Il Protocollo non prevede in alcun modo la standardizzazione della terapia farmacologica e in particolare quella antivirale.

**DATABASE**

Lo studio prevede la creazione di un Database in cui tutti i dati saranno resi anonimizzati. Tale Database sarà compilato a cura del medico sulla base della consultazione della cartella clinica del paziente partecipante allo studio. I seguenti dati verranno raccolti: età del paziente, terapia per COVID-19 in atto e pregressa, altra terapia in atto (farmaci antipertensivi, statine, eparina e/o altri anticoagulanti), sieropositività per HIV, HBV o HCV, presenza di patologia neurologica pregressa e sua tipologia , presenza di sintomi neurologici al momento dell’esecuzione di puntura lombare e sua tipologia, dati di laboratorio (Hb, WBC con n. di linfociti/mm3, neutrofili/mm3, piastrine/mm3, creatinina e azotemia, ALT/AST e bilirubina, D-dimero, Sodio, Potassio, fibrinogeno, PT, PTT, ferritina, LDH, IL-6).

**ASSESSMENT NEUROCOGNITIVO**

L’assessment neurocognitivo avrà lo scopo di osservare in pazienti affetti da SARS/COVID-19 le difficoltà cognitive, in particolare scarse performance attentive, e difficoltà comportamentali (funzioni frontali, disinibizione e discontrollo degli impulsi) ritenute verosimilmente secondarie ad aspetti psicoemotivi di base (flessione del tono dell'umore, disturbi d'ansia, ideazione suicidaria) come esiti dell'isolamento a cui i pazienti sono costretti, e nei casi più gravi quali pazienti sottoposti a rianimazione, la presenza di una condizione post-trauamtica.

1. **Tests Neuropsicologici**

**Stato cognitivo generale:**

- Mini Mental State Examination o MMSE (14) (ALLEGATO B): valutazione dei disturbi dell’efficienza intellettiva. E’ costituito da trenta items che fanno riferimento a sette aree cognitive differenti: orientamento nel tempo, orientamento nello spazio, registrazione di parole, attenzione e calcolo, rievocazione, linguaggio, prassia costruttiva.

1. **Apprendimento e Memoria (ALLEGATO C)**

- Test delle 15 parole di Rey (15) **(ALLEGATO C)**: L’esaminatore legge una lista di 15 parole al ritmo di una parola al secondo per 5 volte, e dopo ogni presentazione richiede la rievocazione delle parole in ordine libero; dopo un intervallo di 15 minuti si richiede una rievocazione differita delle 15 parole.

1. **Velocità di elaborazione delle informazioni**

- Trial Making Test A (TMT A) (17) **(ALLEGATO C)**: Al soggetto viene dato un foglio con sopra prestampati dei numeri, l’esaminatore fornisce la consegna di collegare il più velocemente possibile i numeri in ordine crescente. L’esaminatore avrà cura di correggere il paziente durante l’esecuzione della prova senza interrompere il tempo. Il punteggio è dato dal tempo necessario per completare la prova.
- Wais digit symbol (18) **(ALLEGATO C)**: Viene fornita al soggetto una scheda, in alto si trova una leggenda composta da numeri associati a simboli e in basso una serie di 100 item composti da soli numeri. Si chiede al soggetto di associare il più velocemente possibile i numeri al corrispondente simbolo. Il soggetto ha a disposizione 90 secondi. I primi 7 item sono di prova e non vengono conteggiati nel punteggio finale; per gli altri 93 viene attribuito un punto per ogni risposta corretta.

1. **Attenzione/Memoria a Breve Termine/Memoria di Lavoro:**

- Span di cifre in avanti (19) **(ALLEGATO C)**: L’esaminatore presenta oralmente una sequenza di cifre (crescente) che il soggetto deve ripetere correttamente immediatamente dopo la presentazione. Lo span Verbale in avanti è dato dal numero di item che componevano la cifra più lunga che il soggetto è riuscito a ripetere correttamente.
- Span di cifre indietro (19) **(ALLEGATO C)**: L’esaminatore presenta oralmente una sequenza di cifre (crescente) che il soggetto deve ripetere nell’ordine inverso (partendo dall’ultimo numero e tornando indietro fino al primo) immediatamente dopo la presentazione. Lo span verbale indietro è dato dal numero di item che componevano la cifra più lunga che il soggetto è riuscito a ripetere correttamente.
- Test di Barrage doppio (MFTC) (20) (ALLEGATO D): valutazione spaziale e delle funzioni di attenzione selettiva. Il test si articola sulla ricerca di stimoli costituiti da quadrati con due linee all’interno orientate in modo differente tra molti distrattori visualmente simili.

1. **Funzioni esecutive**

- Fluenza verbale fonemica e categoriale (21) **(ALLEGATO C)**: è una prova di linguaggio che valuta anche la flessibilità cognitiva. Si fornisce al soggetto una lettera dell’alfabeto e gli si chiede di produrre quante più parole gli vengono in mente che iniziano con quella lettera fatta eccezione per i nomi propri. La prova si compone di 3 lettere e si dà un minuto di tempo per lettera. Nella prova categoriale invece, si chiede al soggetto di dire tutte le parole che appartengono ad una data categoria.
- Trial Making Test B (TMT B) (17) **(ALLEGATO C)**: Al soggetto viene dato un foglio con sopra prestampati dei numeri e delle lettere, l’esaminatore fornisce la consegna di collegare il più velocemente possibile i numeri in ordine crescente alternandoli con le lettere in ordine alfabetico. L’esaminatore avrà cura di correggere il paziente durante l’esecuzione della prova senza interrompere il tempo. Il punteggio è dato dal tempo necessario per completare la prova.
- Test di stroop (22) **(ALLEGATO C)**: Il Soggetto deve denominare in 20 sec. (per ogni prova) il colore degli item. Nella prima prova (denominazione) il soggetto ha davanti dei pallini colorati mentre nella seconda prova (interferenza) ha davanti il nome dei colori scritti con inchiostro di colore differente. Ogni prova è costituita da 60 item. Si calcola il punteggio dell’Interferenza dato dalla differenza nel numero di item denominati correttamente in 20 secondi nella prima prova e nella seconda.

1. **Funzioni prassiche/visuo-spaziali:**

- Copia della Figura di Rey-Osterrieth (24 **(ALLEGATO C)**: si presenta al soggetto una figura complessa e gli si chiede di ricopiarla. Nella figura sono riconoscibili 18 elementi che il soggetto deve posizionare correttamente, per ogni elemento si valuta se è posizionato correttamente e se la copia dell’elemento è completa. La figura può essere richiamata a memoria dopo un intervallo di tempo e la rievocazione differita consente la valutazione della memoria a lungo termine visiva.

**2.Questionari e Scale**

1. **Questionario per la valutazione delle abilità nella vita quotidiana**

- *Instrumental Activities of Daily Living-IADL (28* **(ALLEGATO H):** è una breve intervista tesa a valutare le abilità del paziente nella vita quotidiana. Indaga 8 abilità: Abilità ad usare il telefono, fare la spesa, preparare i pasti, cura della casa, fare il bucato, spostamenti fuorcasa, assunzione dei propri farmaci, uso del proprio denaro. Tale strumento è utile ai fini della differenziazione nell’esecuzione degli items i quali richiedono una complessa organizzazione neuropsicologica regolata dalle funzioni cognitive: la loro valutazione e soprattutto le variazioni longitudinali possono essere test di declino cognitivo di semplice esecuzione.

1. ***Questionari e scale per la valutazione dei sinto*mi Neuropsichiatrici**

- *Beck DepressionInventory II (BDI) (29, 30)* **(ALLEGATO I)***.* Il BDI-II è uno strumento self-report che consente di valutare la presenza e l’intensità dei sintomi depressivi. Il test, composto da 21 item, restituisce un punteggio totale e due punteggi relativi alle aree: -**Somatico-Affettiva**, che riguarda le manifestazioni somatiche-affettive della depressione quali perdita di interessi, perdita di energie, modificazioni nel sonno e nell’appetito, agitazione e pianto, ecc. **-Cognitiva**, che riguarda le manifestazioni cognitive quali pessimismo, senso di colpa, autocritica, ecc.
- *Beck Anxiety Inventory (BAI) (31, 32* **(ALLEGATO I)***.* Il BAI è uno strumento self-report che permette di valutare la gravità della sintomatologia ansiosa negli adulti. Il questionario si compone di 21 item (descrizioni di sintomi di ansia somatica, soggettiva o correlata a fobie), da valutare su una scala a quattro punti (da 0 a 3).
- *PittsburgSleep Quality Index — PSQI (33)* **(ALLEGATO L)***.* È una scala di autovalutazione si compone di 19 item, raggruppati in 7 item compositi, valutati su una scala da 0 a 3, che sommati danno il punteggio globale del PSQI, che può andare da 0 a 21. Questi 7 item compositi rappresentano la qualità soggettiva del sonno, la latenza di sonno, la durata del sonno, l’efficacia abituale del sonno, i disturbi del sonno, l’uso di farmaci ipnotici ed i disturbi durante il giorno.

**PROCEDURE DI LABORATORIO**

1. **Valutazione dell’attivazione immunitaria e della presenza di fattori proinfiammatori**

I biomarcatori di interesse, verranno analizzati mediante differenti metodologie: Luminex assay, che permette il dosaggio simultaneo di più analiti, per le citochine pro-infiammatorie (IL-6, IL-1B, IL-8, TNFalfa, MIP-1B, MCP-1, RANTES, GM-CSF, G-CSF), citochine Th1 (IL-2, IL-12p70, IFNgamma), Th2 (IL-4, IL-13, IL-10, IL-5) e Th17 (IL-17, IL-19) e saggio ELISA(diretto o competitivo) per Neopterina e sCD14 e NF-L. Per la valutazione dell’attivazione B saranno quantificate le catene libere leggere kappa e labda mediante nefelometria.

1. **Caratterizzazione dei linfociti T, B e NK nel sangue periferico e nel CSF**

La caratterizzazione dei linfociti T, B e NK verrà effettutata mediante citofluorimetria a flusso multiparametrica utilizzando dei tubi altamente standardizzati. Attraverso l’utilizzo di anticorpi monoclonali specifici, le cellule del sangue periferico e del CSF saranno così definite: linfociti T (CD45+CD3+), linfociti CD4 (CD45+CD3+CD4+), linfociti CD8 (CD45+CD3+CD8+), linfociti B (CD45+CD3-CD19CD20+), cellule NK (CD45+CD3-CD19-CD20-CD56+). Inoltre, l’attivazione dei linfociti T sarà valutata mediante analisi del marcatore CD38 e la clonalità dei linfociti B sarà valutata mediante analisi dell’espressione delle catene kappa e lambda. Infine, l’espressione del CD5 permetterà di valutare la presenza di popolazioni immature. Le cellule saranno acquisite mediante FACS Canto II e analizzate mediante software Diva.

1. **Analisi del danno di barriera ematoencefalica**

Saranno dosati, mediante metodi automatizzati di laboratorio (nefelometria), i valori di Albumina e le immunoglobuline di tipo IgG nelle coppie plasma/liquor e successivamente sarà determinato il contestuale rapporto in maniera da ottenere l’indice di Link.

1. **Valutazione della presenza di SARS-CoV-2 RNA nelle coppie plasma/liquor e valutazione della risposta anticorpale specifica**

I livelli di SARS-CoV-2 RNA nel plasma e nel liquor saranno determinati in real time RT-PCR mediante l’impiego di test diagnostici disponibili in commercio secondo le linee guida emanate dall’OMS.L’RNA virale sarà sottoposto a sequenziamento per determinare l’eventuale presenza di mutazioni rispetto al virus isolato nel distretto respiratorio.

Per quanto riguarda invece la determinazione della risposta anticorpale specifica per SARS-CoV-2 sarà analizzata mediante immunofluorescenza indiretta (IFA) la presenza di IgA, IgM ed IgG sia nel compartimento plasmatico che in quello liquorale ovvero immunoenzimatica.

**PROCEDURE DI NEUROIMAGING**

1. **Valutazione di eventuale alterazione metabolica di specifiche aree encefaliche**

Le aree cerebrali specificamente quelle della corteccia frontale e prefrontale ovvero della zona limbica saranno studiate mediante RM funzionale dell’encefalo (fMR) che permetterà di documentare il consumo di ossigeno da parte delle cellule neuronali per ciascuna delle aree di interesse, permettendo di correlare lo stesso al grado di attivazione neuronale. Lo studio fMR sarà completato con studio morfologico in condizioni di base, con valutazione dei coefficienti di diffusione e di diffusione apparente, e dopo perfusione di mdc paramagnetico per documentare eventuali danni di barriera ematoencefalica.

1. **Valutazione di danno perfusionale a livello di specifiche aree encefaliche**

Il danno del microcircolo cerebrale legato a SARSCOV2 sarà studiato mediante esame TC encefalo con mdc con studio perfusionale su strato selezionato o in alternativa con RM perfusionale nei pazienti con adeguata compliance.

**ASPETTI ETICI E TUTELA DELLA PRIVACY**

Lo studio sarà presentato al Comitato Etico dell’INMI “L. Spallanzani” e verrà messo in atto solo dopo la sua piena approvazione. Prima dell’inclusione dei pazienti nello studio i medici coinvolti dovranno spiegare ai potenziali soggetti il protocollo di studio e le implicazioni associate alla loro partecipazione, illustrando le Informazioni per il Paziente ed il Consenso Informato (ALLEGATO A, B).

Tutte le informazioni connesse alla partecipazione al presente programma saranno trattate in modo strettamente riservato in conformità alle norme di buona pratica clinica (Decreto Ministero della Sanità aprile 99 ed integrazioni), nonché a quelle per la tutela delle persone e di altri soggetti rispetto al trattamento dei dati personali (D.Lgs. 196/03). In particolare, ai sensi e per gli effetti dell’art. 3, primo comma, del D.Lgs. 196/03, i dati personali (inclusi i dati “sensibili”) saranno registrati, elaborati, gestiti e archiviati - in forma cartacea e informatizzata - per le esclusive finalità connesse all’espletamento del presente programma.

Il medico curante fornirà al paziente le informazioni relative allo studio e raccoglierà il Consenso Informato. Al momento dell’arruolamento nello studio a ciascun partecipante sarà assegnato un codice anonimo la cui corrispondenza con le generalità della paziente è nota esclusivamente al medico curante. I dati saranno successivamente soggetti ad elaborazione statistica e i risultati delle analisi, costituiti da dati aggregati, saranno inseriti in pubblicazioni scientifiche e/o presentazioni a congressi. I risultati del presente studio verranno presentati sotto forma di abstract e presentazioni a Congressi Nazionali e Internazionali. E’ prevista la stesura di lavori *in estenso* da sottomettere a riviste con *peer review*.

**FONTE DEI FINANZIAMENTI**

I finanziamenti dedicati alle differenti analisi da eseguire sulle coppie plasma/liquor e sugli esami di *neuroimaging* incluse nello studio saranno opportunamente individuati e destinati *ad hoc* per ciascun progetto di studio disegnato.

**RICADUTA SUL SISTEMA SANITARIO NAZIONALE**

La dimostrazione di un coinvolgimento virologico da parte di SARSCOV2 del Sistema Nervoso Centrale potrebbe essere associato ad un’infiammazione acuta o subacuta di tale compartimento alla base di una compromissione neurologica, anche in pazienti pauci-sintomatici, con conseguente comparsa di deficit neurologici funzionali e/o anatomici non altrimenti caratterizzabili ovvero con conseguente peggioramento della funzione respiratoria da mancato controllo centrale. Lo studio di tale fenomeno permetterebbe di disegnare strategie diagnostiche e terapeutiche capaci di intercettare la patologia neurologica in fase precoce, sulla base dell’individuazione di specifici fattori predittivi di patologia.

**BIBLIOGRAFIA**

1. <https://www.ecdc.europa.eu/en/covid-19-pandemic2>.
2. <http://www.protezionecivile.gov.it/attivita-rischi/rischio-sanitario/emergenze/coronavirus>
3. <http://www.salute.gov.it/portale/nuovocoronavirus/dettaglioContenutiNuovoCoronavirus.jsp?lingua=italiano&id=5338&area=nuovoCoronavirus&menu=vuoto>
4. Dubé et al. J Virol, 2018, 92, e00404-18
5. Cheng SKW et al. B J Psych, 2004, 184, 359-360

6. Morfopoulou et al. N Engl J Med, 2016, 375, 497-8

7. Lau et al, Emerg Inf Dis., 2004, 10,342-344

8. Baig Am, ACS Chemical Neurosciences, 2020

9. Lechien JR, et al, 2020 Apr 6Eur Arch Otorhinolaryngol. 2020

10. Li YC,et al. J Med Virol. 2020. Review

11. Mao L et al., MedRxiv 2020

12. Poyiadji N, et al . Reviews and Commentary,Images in Radiology; 30Mar20;

13. Filatov A, et al. Cureus 12(3): e7352

14. Measso G, et al. Developmental Neuropsycol 1993;9:77-85.

15. Carlesimo G, et al. Archivio di Psicologia, Neurologia e Psichiatria, 1995,56(4), 471-488.

16. Novelli G, et al. Archivio di Psicologia, Neurologia e Psichiatria, 1986,47(2), 278-296.

17. Amodio P, et al. J Hepatol 2008;49:346-353

18.Orsini A, et al. Firenze: Giunti O.S. Organizzazioni Speciali,1997.

19. Monaco M, et al. Neurol Sci. 2013 May;34(5):749-54.

20. Marra C, et al. Neurol Sci. 2012 Feb 19

21. **Marra C, et al. Neuropsychology. 2007 Mar;21(2):207-11.**

22.Valgimigli S, et al. Giornale Italiano di. Psicologia, 37 (4), 945-953.

23. Laiacona M, et al. Neurological Sciences. 2000 Nov 21(5):279.

24. Carlesimo A, et al. Nuova Rivista di Neurologia 12:1–13 6.

25.Carlesimo G, et al. Eur Neurol. 1996;36(6):378-84

26. Nucci M, et al. Aging clinical and experimental research, 24, 218-126.

27. Nucci M, et al. Giornale Italiano di Psicologia, 1, 155-174.

28. Lawton, et al. The Gerontologist, 9(3), 179-186.

29. Beck A, et al. Beck Depression Inventory. 2nd edition. San Antonio, Tex, USA: The Psychological Corporation; 1996.

30. Ghisi M, et al. Beck Depression Inventory. 2nd edition. Firenze, Italy: Organizzazioni Speciali; 2006

31. Beck AT, et al. Beck anxiety inventory manual. San Antonio, TX: Psychological Corporation. 1990

32. Sica C, et al. L’adattamento italiano del BAI. In A.T.Beck & R.A. Steer. Beck Anxiety Inventory. Firenze: Organizzazioni Speciali.2006

33. Curcio G, et al. Neurol Sci. 2013 Apr;34(4):511-9.

34. Posner K. Am J Psychiatry. 2011;168(12):1266-1277.

35. Apolone G, Journal of clinical epidemiology. 1998. 51 (11), 1025–1036.

36. Apolone G, 2015. Milano, It.

37. Rabin R, et al. Ann. Med. 2001; 33:337-343.

38. Sica C, et al. Dipartimento di Psicologia - Università degli Studi di Firenze. Dipartimento di Psicologia Generale – Università degli Studi di Padova. “Coping Orientation to Problems Experienced-Nuova Versione Italiana (COPE-NVI): uno strumento per la misura degli stili di coping.
